# The analgesic effects of buprenorphine (Vetergesic or Simbadol) in combination with carprofen in dogs undergoing ovariohysterectomy: a randomized, blinded, clinical trial

**DOI:** 10.1186/s12917-018-1628-4

**Published:** 2018-10-05

**Authors:** Ryota Watanabe, Beatriz P. Monteiro, Marina C. Evangelista, Amélie Castonguay, Daniel Edge, Paulo V. Steagall

**Affiliations:** 1 Department of Clinical Sciences and Animal Pharmacology Research Group of Quebec (GREPAQ; Groupe de recherche en pharmacologie animale du Québec), Faculty of Veterinary Medicine, Université de Montréal, 3200 rue Sicotte, Saint-Hyacinthe, QC J2S 2M2 Canada; 2 Zoetis Inc, Parsippany, NJ USA

**Keywords:** Analgesia, Buprenorphine, Canine, Ovariohysterectomy, Pain, Simbadol, Vetergesic

## Abstract

**Background:**

Buprenorphine is a potent lipophilic opioid analgesic that is largely used in the multimodal treatment of acute pain. Simbadol (buprenorphine hydrochloride) is the first and only FDA-approved high-concentration formulation of buprenorphine for use in cats. The aim of this study was to evaluate the analgesic efficacy of carprofen in combination with one of two commercial formulations of buprenorphine (Simbadol and Vetergesic, 1.8 mg/mL and 0.3 mg/mL, respectively) in dogs undergoing ovariohysterectomy. Twenty-four dogs were included in a randomized, prospective, controlled, clinical trial. Patients were randomly divided into 2 groups as follows. Dogs were premedicated with acepromazine (0.02 mg/kg) and either 0.02 mg/kg of Vetergesic or Simbadol intramuscularly (Vetergesic group – VG; Simbadol group – SG, respectively; *n* = 12/group). General anesthesia was induced with propofol and maintained with isoflurane in 100% oxygen. Carprofen (4.4 mg/kg SC) was administered after induction of anesthesia. Heart rate, respiratory rate, blood pressure, pulse oximetry, pain scores using the Glasgow Composite Pain Scale Short Form (CMPS-SF), sedation scores using a dynamic interactive visual analogue scale and adverse events were evaluated before and after ovariohysterectomy by an observer who was unaware of treatment administration. If CMPS-SF scores were ≥ 5/20, dogs were administered rescue analgesia (morphine 0.5 mg/kg IM). Statistical analysis was performed using linear mixed models and Fisher’s exact test (*p* < 0.05).

**Results:**

Pain and sedation scores and physiological parameters were not significantly different between treatments. Three dogs in VG (25%) and none in SG (0%) required rescue analgesia (*p* = 0.109). Adverse effects (i.e. vomiting and melena) were observed in two dogs in SG and were thought to be related to stress and/or nonsteroidal anti-inflammatory drug toxicity.

**Conclusions:**

The administration of buprenorphine with carprofen preoperatively provided adequate postoperative analgesia for the majority of dogs undergoing OVH without serious adverse events. Prevalence of rescue analgesia was not significantly different between groups; however, it could be clinically relevant and explained by a type II error (i.e. small sample size). Future studies are necessary to determine if analgesic efficacy after Simbadol and Vetergesic is related to individual variability or pharmacokinetic differences.

## Background

Ovariohysterectomy (OVH) is commonly performed in dogs and results in postoperative pain that is associated with behavioral changes [1, 2]. Safe and effective pain management is important in patient care and new analgesic techniques are constantly evolving to address this need. Buprenorphine is a potent semisynthetic highly lipophilic opioid analgesic that is largely used in the multimodal treatment of acute pain. The drug has a complex pharmacologic profile but is generally considered as a partial mu opioid agonist [3, 4]. Buprenorphine causes negligible cardiovascular effects and it is used for the treatment of mild to moderate pain such as ovariohysterectomy (OVH) in dogs and cats because of its long-lasting analgesic properties and few adverse-effects [4–6]. Indeed, a dose of 0.02 mg/kg of buprenorphine combined with a non-steroidal anti-inflammatory drug (NSAID) has been recommended for postoperative analgesia in dogs undergoing ovariohysterectomy [7, 8].

There are different commercial products of buprenorphine in the market with concentrations of 0.3 mg/mL (e.g. Vetergesic; buprenorphine hydrochloride; Champion Alstoe, Whitby, ON, Canada). On the other hand, Simbadol (buprenorphine hydrochloride, Zoetis, Parsippany, New Jersey, USA) has a concentration of 1.8 mg/mL and is the first and only FDA-approved opioid analgesic for use in cats to provide 24-h postoperative pain control after a single dose and can be administered for up to 3 days [9]. There is currently a clinical interest in using Simbadol for postoperative pain relief in dogs with the current opioid shortage in veterinary medicine in the United States [10]. In addition, it is not known whether different commercial products of buprenorphine could be used interchangeably or if drug concentrations would impact postoperative analgesia.

The aim of this study was to evaluate the analgesic efficacy of carprofen in combination with one of two commercial formulations of buprenorphine (Simbadol or Vetergesic) in dogs undergoing ovariohysterectomy. In addition, the physiological and adverse events produced by the two treatments were recorded. Our hypothesis was that the administration of Simbadol or Vetergesic with carprofen would produce similar postoperative pain scores and prevalence of rescue analgesia without serious adverse events that would require medical treatment.

## Results

Age, body weight, body condition score, hematocrit, total protein, surgery and anesthesia times, and time to extubation are presented in Table 1. Surgery and anesthesia times were significantly longer in SG when compared with VG (*p* = 0.032 and *p* = 0.028, respectively).

**Table 1:** Demographic data, surgery and anesthesia time, and time to extubation of dogs undergoing ovariohysterectomy

Variable	Vetergesic (*n* = 12)	Simbadol (n = 12)	*p* value
Body weight (kg)	12.1 (9.7)	19.3 (10.9)	0.219
Body condition score (1–9)	5 (4–5)	5 (4–6)	0.378
Age (years)	2.7 (2)	4.2 (3)	0.319
Hematocrit (%)	40.4 (4.5)	41.6 (2.3)	0.378
Total protein (g/dL)	6.3 (0.75)	6.7 (0.79)	0.216
Surgery time (min)	34.8 (8.9)	44.9 (12.4)	0.032
Anesthesia time (min)	47.8 (8.4)	56.7 (12)	0.028
Time to extubation (min)	8.3 (3.4)	8.5 (2.9)	0.849

Dogs treated with carprofen in combination with two concentrations of buprenorphine (Vetergesic or Simbadol). Values are expressed as mean (SD) with the exception of body condition score which is reported as median (range)

### Physiological parameters

Physiological parameters are presented in Table 2. There were no significant differences between treatment groups.

**Table 2:** Pain and sedation scores and physiological parameters in dogs undergoing ovariohysterectomy

Time points	Drugs	CMPS-SF	DIVAS	Temperature (°C)	HR (bpm)	RR (bpm)	SAP (mmHg)	MAP (mmHg)	DAP (mmHg)	SpO2 (%)
Time 0 (baseline values)	VG	0 (0)	0 (0)	38.5 (38.2–38.8)	125 (107–143)	37 (30–44)	159 (141–177)	116 (104–127)	91 (79–102)	97 (96–98)
	SG	0 (0)	0 (0)	38.5 (37.9–39.0)	117 (105–129)	37 (30–44)	167 (145–188)	123 (111–134)	100 (89–110)	96 (95–98)
15 min after premedication	VG		22.3 (18.4–26.3)	38.2 (38.1–38.3)^a^	94 (80–108)^a^	34 (28–40)	146 (125–166)	100 (89–111)^a^	75 (67–84^a^	98 (97–99)
	SG		23.7 (13.4–33.9)	38.1 (37.9–38.3)^a^	100 (86–115)	34 (27–40)	140 (133–146)^a^	97 (90–104)^a^	77 (70–83) ^a^	96 (95–97)
Postoperative 0.5 h	VG	1.25 (0.6–1.9)	38.9 (28.1–49.7)	37.0 (36.7–37.4)^a^	102 (84–120)^a^	34 (30–38)	157 (143–172)	117 (104–130)	95 (84–106)	97 (96–97)
	SG	0.92 (0.4–1.4)	38.1 (24.2–52)	36.8 (36.5–37.1)^a^	109 (98–121)	29 (24–34)	161 (153–170)	118 (113–124)	95 (89–101)	97 (96–97)
Postoperative 1 h	VG	1.5 (0.4–2.6)	27.8 (16,5–39)	37.1 (36.8–37.5)^a^	94 (76–112)^a^	31 (25–37)	158 (145–172)	114 (102–126)	87 (79–96)	97 (96–98)
	SG	1 (0.6–1.4)	32.7 (19.9–45.5)	37 (36.7–37.3)^a^	105 (94–116)	31 (27–36)	153 (141–164)^a^	115 (109–122)	93 (87–99)	96 (95–97)
Postoperative 2 h	VG	1.27 (−0.1–2.7)	18.9 (15.5–22.4)	37.2 (36.9–37.6)^a^	91(75-108)^a^	29 (24–35)	151 (136–165)	105 (95–115)	83 (74–92)	97 (96–99)
	SG	0.5 (0.1–0.9)	22 (16.3–27.4)	37.2 (36.9–37.5)^a^	103 (89–118)	31 (27–35)	161 (153–169)	115 (109–121)	90 (82–99)	96 (95–97)
Postoperative 3 h	VG	1 (−0.3–2.3)	13.9 (11.3–16.5)	37.4 (37.1–37.6)^a^	92 (68–116)^a^	31 (26–35)	157 (143–170)	108 (100–116)	82 (76–88)	97 (96–98)
	SG	0.7 (0.3–1.1)	15.3 (11.2–19.3)	37.4 (37.3–37.6)	88 (79–97)^a^	32 (27–36)	156 (147–166)	113 (108–119)	89 (84–95)	97 (96–98)
Postoperative 4 h	VG	0.9 (0.3–1.5)	10.4 (6.5–14.4)	37.4 (37.2–37.6)^a^	86 (66–105)^a^	32 (27–37)	157 (139–176)	116 (105–127)	90 (82–98)	97 (96–99)
	SG	0.5 (0.1–0.9)	12.3 (7.9–16.6)	37.5 (37.4–37.7)^a^	88 (77–99)^a^	32 (27–36)	159 (149–169)	110 (104–116)^a^	85 (79–90)^a^	96 (95–97)
Postoperative 6 h	VG	0.3 (−0.4–1.1)	6.8 (3.8–9.8)	37.6 (37.4–37.8)^a^	92 (74–109)^a^	31 (24–39)	155 (138–172)	109 (99–119)	82 (73–91)	97 (96–98)
	SG	0.2 (−0.1–0.4)	6.8 (4.4–9.1)	37.6 (37.4–37.9)^a^	96 (85–107)^a^	33 (28–37)	160 (148–171)	114 (106–122)	90 (84–96)	97 (96–97)
Postoperative 8 h	VG	0.1 (−0.2–0.4)	4.1 (0.8–7.3)	37.8 (37.5–38.0)^a^	89 (78–100)^a^	31 (26–37)	158 (139–178)	107 (96–118)	82 (73–91)	97 (96–99)
	SG	0.1 (−0.1–0.3)	3.3 (2.1–4.4)	37.9 (37.7–38.0)^a^	101 (87–114)	33 (29–38)	159 (146–172)	110 (110–120)^a^	88 (79–96)^a^	97 (96–98)

Mean (CI) for pain scores using the Glasgow Composite Pain Scale short-form (CMPS-SF), sedation scores using the dynamic and interactive visual analogue scale (DIVAS) and physiological parameters including temperature, heart rate (HR), respiratory rate (RR), systolic (SAP), mean (MAP) and diastolic (DAP) blood pressure, and pulse oximetry (SpO_2_) in dogs undergoing ovariohysterectomy and treated with carprofen in combination with two concentrations of buprenorphine (Vetergesic - VG or Simbadol - SG)

^a^Significant difference when compared with baseline values

### Sedation scores

There were no significant differences between treatments. In both groups, DIVAS was significantly higher 15 min after premedication, and at 0.5, 1, 2, 3, 4 and 6 h when compared with baseline values (*p* < 0.001 for all time points) (Table 2).

### Pain scores

There were no significant differences between treatments. Pain scores were significantly higher at 0.5, 1 (*p* < 0.001) and 4 h (*p* = 0.002) in VG and at 0.5 h (*p* = 0.002) in SG when compared with baseline values (Table 2). Rescue analgesia was administered to three dogs in VG (3/12 dogs; 25%), and none in SG (0/12 dogs; 0%). Prevalence of rescue analgesia was not significantly different between treatment groups (*p* = 0.109). Dogs in VG that required rescue analgesia had variable body weights (21.9, 3.6 and 2.4 kg) and the same body condition score (5).

### Adverse events

In SG, one dog presented vomiting and melena and another dog presented melena at 8 h postoperatively. Physical and clinical pathology examinations (complete blood count and serum biochemistry profile) were unremarkable in these two dogs. Maropitant (1 mg/kg, 10 mg/mL, Cerenia; Zoetis, Kirkland, QC, Canada by the subcutaneous route) was administered once in the dog with vomiting and melena; omeprazole (0.5 mg/kg/SID, 10 mg/tab, Losec, AstraZeneca, ON, Canada) and metronidazole (13 mg/kg/BID, 250 mg/tab, AA pharma inc., Toronto, ON, Canada) were administered for 7 days in both dogs. Adverse events were not recorded again and both dogs fully recovered within 12–24 h upon return to their shelter facilities. Total number of dogs that developed an adverse event was not significantly different between treatment groups (*p* = 0.239).

Hypothermia (defined as rectal temperature below 36.5 °C) [11] was observed in two dogs in VG and three dogs in SG at 0.5 h, in one dog in VG and two dogs in SG at 1 h, and in one dog in each group at 2 h. Prevalence of hypothermia was not significantly different between groups (times 0.5 and 1 h: *p* = 0.5; time 2 h: *p* = 0.761).

## Discussion

This study demonstrated that the intramuscular administration of Simbadol provides effective postoperative analgesia in combination with carprofen in dogs undergoing OVH. Pain scores and prevalence of rescue analgesia were not significantly different between SG and VG; however, one may argue that differences in rescue analgesia could be of clinical relevance. The observed analgesic effect was different between treatments (prevalence of rescue analgesia: 25% of dogs in VG versus 0% dogs in SG), albeit not statistically significant. Had one more dog been administered rescue analgesia in VG, the difference would have been significant between treatments. Indeed, the lack of difference between groups could be related to a type II error due to small sample size suggesting a clinically relevant finding masked by potential low statistical power. On the other hand, this finding is difficult to explain because the only difference between these two commercial products is the concentration of buprenorphine (1.8 and 0.3 mg/mL for SG and VG, respectively) which results in different final volumes of injection for the same dose. The rate of diffusion of a compound across a membrane depends on its concentration gradient across membranes, liquid/partition coefficient and diffusion coefficient of the drug (Fick’s law of diffusion) [12]. In this study, the rate of diffusion should be similar for Simbadol and Vetergesic because the same active ingredient, dose, route of administration and body location for injection were used; excipients are similar between these two drugs. Contrarily, a study in cats showed that the concentration of buprenorphine (0.3, 0.6 and 1.2 mg/mL) influenced maximum plasma concentrations but not time to peak effect or thermal antinociception [13]. If the same findings were corroborated in this study, higher plasma concentrations of buprenorphine in SG would allow the drug to transfer down the concentration gradient into the central nervous system allowing the drug to occupy more opioid receptors and to produce greater analgesic effect [14]. A pharmacokinetic study is necessary to determine whether these differences are a) due to absorption, distribution, metabolism or elimination, or if b) perhaps these three dogs in VG had lower nociceptive thresholds for pain or c) buprenorphine was not the best opioid choice for these individuals. In any of these scenarios, rescue analgesia would have been administered even if these three dogs had been allocated to SG instead of VG. Finally, dogs in SG were heavier than in VG. It is possible that dogs in VG could have received a smaller dose than SG if doses had been based on body surface area. In this case, treatments would not have been equivalent. However, labelled dose recommendations for buprenorphine do not take in consideration body surface area in veterinary medicine.

Pain scores were also not significantly different between VG and SG. This result is not surprising because scores were excluded from statistical analysis following administration of rescue analgesia. This approach may overestimate the analgesic effect of a treatment because higher pain scores are possibly omitted, and selection bias is introduced while avoiding analysis bias. This approach limits the ability to detect significant differences among treatments using pain scores; however, this should not be an issue when prevalence of rescue analgesia is used as the main outcome of a clinical trial [15].

Surgery and anesthesia time were significantly longer in SG when compared with VG most likely due to differences in mean body weight. Dogs in SG were heavier than VG even if not significantly different and OVH would naturally take more time in the first than the latter group. Even so, surgery and anesthesia times were shorter in this study than previous similar reports [7, 8].

Some physiological parameters including HR, SAP, MAP and DAP were significantly lower after premedication than baseline values. Acepromazine produces peripheral vasodilation and reduces blood pressure due to antagonism of α1- adrenergic receptors. Decreases in HR and blood pressure may be observed after the administration of buprenorphine due to increased vagal stimulation [6]. Sedation and decreases in catecholamine concentrations via dopaminergic effect may result in lower HR after premedication and these results are not surprising. Physiological values in the current study were within normal ranges and these changes were not clinically relevant [4, 16]. Both concentrations of buprenorphine did not induce important cardiorespiratory changes in dogs undergoing OVH. Hypothermia was observed in some dogs in both groups up to 2 h postoperatively. Decreases in temperature are commonly observed after general anesthesia and its prevalence was not statistically different between groups.

Vomiting and/or melena were observed in two dogs in SG at 8 h postoperatively. Main differential diagnosis for these gastrointestinal clinical signs include stress, primary gastrointestinal disease and NSAID toxicity.

Gastrointestinal toxicity is recognized as one of the most common signs of NSAID toxicity and they could be related to individual sensitivity to NSAID administration or an idiopathic reaction [17]. However, carprofen has been administered in several studies for acute pain management without clinically relevant adverse events [17]. It may be also possible that dogs did not have enough time to acclimate to the hospital setting after transportation presenting with stress-induced gastrointestinal disorder. To the authors’ knowledge, these clinical adverse effects have not been reported after the administration of buprenorphine in dogs and were not considered to be treatment- (Vetergesic or Simbadol) related.

## Conclusion

The administration of carprofen with either Simbadol or Vetergesic preoperatively provided adequate postoperative analgesia for the majority of dogs undergoing OVH and without serious adverse events. Prevalence of rescue analgesia was not significantly different between groups; however, it could be clinically relevant and explained by a type II error (i.e. small sample size). Future studies are necessary to determine if analgesic efficacy after Simbadol and Vetergesic is related to individual variability or pharmacokinetic differences.

## Methods

This study was a prospective, randomized, blinded, controlled, clinical trial conducted at Université de Montréal. The study was approved by the animal care committee of the Université de Montréal (16-Rech-1846). This study follows The Consolidated Standards of Reporting Trials (CONSORT) [18].

### Animals

Twenty-four adult female dogs from shelter facilities were enrolled to undergo OVH. Dogs were included if they were considered healthy based on medical history, complete physical examination and hematocrit and total protein. Dogs had to be up to date on vaccination and parasite control. Exclusion criteria included aggression, anxiety, pregnancy or any sign of disease. Dogs were admitted approximately 16 h before surgery. Food but not water was withheld for 8–12 h.

### Anesthetic protocol, surgery and treatments

Dogs were randomly allocated in one of two groups (Vetergesic group – VG or Simbadol group – SG) (*n* = 12/group). Randomization was performed by an individual not involved in pain assessment using a random permutation generator (www.randomization.com). Premedication was performed with acepromazine (0.02 mg/kg; Acepromazine maleate, Gentès & Bolduc, Saint-Hyacinthe, QC, Canada) and either 0.02 mg/kg of Vetergesic (VG) or Simbadol (SG) by the intramuscular route of administration (i.e. epaxial muscles). Approximately 20 min later, an intravenous catheter was aseptically introduced in a cephalic vein and induction of anesthesia was performed with intravenous administration of propofol (10 mg/mL, Propoflo 28, Zoetis, Kirkland, QC, Canada) to effect. After intubation with an appropriately sized endotracheal cuffed tube, dogs were maintained with isoflurane (Isoflurane USP, Fresenius Kabi, Toronto, ON, Canada) in 100% oxygen, and received carprofen (4.4 mg/kg; 50 mg/mL, Rimadyl, Zoetis, Kirkland, QC, Canada) by the subcutaneous route approximately five minutes after anesthetic induction. Anesthetic monitoring was performed according to previously published guidelines [19]. OVH was performed by the same veterinarian with previous experience in surgery. A ventral midline incision was made through the skin, subcutaneous tissue and the aponeurosis of the rectus abdominis muscle and a modified 2-clamp technique was employed. The abdominal wall and subcutaneous tissues were closed using simple continuous pattern of absorbable sutures. The skin was closed using simple interrupted pattern of non-absorbable suture. Surgery time (time elapsed from the first incision until placement of the last suture), anesthesia time (time elapsed from induction of propofol to turning off the vaporizer dial) and time to extubation (time elapsed from turning off the vaporizer dial until extubation) were recorded.

### Data collection

Evaluations were performed before premedication which was approximately 60 mins prior to the induction of anesthesia (time 0, baseline), 15 min after premedication and at 0.5, 1, 2, 3, 4, 6 and 8 h after the end of surgery by an observer who was unaware of treatment administration.

#### Physiological parameters

Temperature, heart rate (HR), respiratory rate (RR), systolic (SAP), mean (MAP) and diastolic (DAP) blood pressure and pulse oximetry (SpO_2_) were recorded. Temperature was measured using a rectal thermometer. HR and RR were recorded via thoracic auscultation. SAP, MAP and DAP were obtained via a non-invasive oscillometric blood pressure device (petMAP, Ramsey Medical Inc., Tampa, FL, USA). The cuff was positioned proximal to the carpus and cuff size was chosen according to the manufacture’s direction. Blood pressure was measured at a level of right atrium three times at each time point and average values were used [20]. SpO_2_ was measured using a pulse oximeter (Rad-5 V, Masimo, Irvine, CA, USA). The probe was placed on the skin between the digits of limbs or over the ears.

#### Sedation scores

Sedation scores were evaluated using the dynamic and interactive visual analogue scale (DIVAS) where 0 was considered as no sedation and 100 as maximum sedation at the aforementioned time-points [21].

#### Pain scores

The Glasgow Composite Pain Scale short-form (CMPS-SF) was used to evaluate pain at the aforementioned time points with the exception of 15 min after premedication. The CMPS-SF is a validated instrument for use in measuring acute pain in dogs [22, 23]. It includes 30 descriptor options within six behavioral categories. Within each category, the descriptors are ranked numerically according to their associated pain. The maximum pain score is achieved with 24 points. For this study, lameness scores (section B of the CMPS-SF) were not included in the evaluation since some dogs could not ambulate due to residual effects of anesthesia. Therefore, rescue analgesia (morphine 0.5 mg/kg, 10 mg/mL, Morphine Sulfate Injection, Sandoz Canada Inc., Boucherville, OC, Canada via intramuscular route of administration) was provided if CMPS-SF scores were ≥ 5/20. For scoring, the dogs were initially evaluated inside their cages without being disturbed. Pain and sedation scores and physiological data were discarded after rescue analgesia and not included in the statistical analysis to avoid bias. However, all dogs were evaluated until the end of the study.

#### Adverse events

Adverse event was defined as any undesirable experience/observation (expected or not) that occurred after administration of the test items whether considered or not to be related to the product [24].

#### Statistical analyses

Statistical analyses were performed using standard statistical software (SPSS Statistics V25, IBM, Armonk, NY, USA). Power analysis was calculated before the study and indicated that a minimum sample size of 8 dogs per group would be needed to detect a difference of 3 points between the 2-means using CMPS-SF and considering an alpha value of 0.05, a power of 80% and a standard deviation within group of 2 points [25]. Data were tested for normality using a Shapiro-Wilk test. Demographic data for each treatment group were compared using independent t-test or Mann-Whitney U test where appropriate. All physiological parameters, DIVAS sedation and CMPS-SF were compared between treatments and time points using a linear mixed model for repeated measures. Time point and treatment group, and their interaction were considered as fixed effects. Dog was considered a random effect and body weight was added as a covariate to the model. The best structure of the covariance was assessed using information criteria that measured the relative fit of a competing covariance model. The Benjamini–Hochberg procedure was used to adjust for multiple comparisons. Total number of rescue analgesia and prevalence of adverse events were compared between treatment groups using Fisher’s exact test. Values of *p* < 0.05 were considered statistically significant.
